# Assessment of lipid profile abnormalities and associated biochemical markers among cardiovascular disease patients in Hajjah Governorate, Yemen: A case-control study

**DOI:** 10.1016/j.ahjo.2026.100829

**Published:** 2026-07-01

**Authors:** Yahya Ali Abdullah Alqadhi

**Affiliations:** a Department of Physiology, Faculty of Medicine and Health Sciences, Hajjah University, Hajjah, Yemen

**Keywords:** Dyslipidemia, Cardiovascular disease, Cardiorenal biomarkers, Hajjah-Yemen, Khat chewing, Logistic regression

## Abstract

**Background:**

Cardiovascular diseases (CVDs) remain a leading cause of morbidity in Yemen. This study evaluates integrated lipid, renal, and hepatic function profiles in CVD patients to provide clinical insights into multi-organ metabolic associations in a resource-limited setting.

**Methods:**

This hospital-based, case-control study at Al-Gomhouri Hospital, Hajjah, Yemen, enrolled 320 participants (160 CVD patients and 160 age- and sex-matched controls). Assessments included lipid profiles, renal markers (uric acid, creatinine), and hepatic markers (albumin, total protein). Multivariate logistic regression identified factors associated with CVD status.

**Results:**

CVD patients exhibited severe atherogenic dyslipidemia, with significantly higher Total Cholesterol (269.7 ± 43.83 mg/dL) and Triglycerides (246.0 ± 58.97 mg/dL) than controls (*P* < 0.001). Markers of organ stress were pronounced in the CVD group, notably higher Serum Uric Acid (7.59 ± 0.95 vs. 3.9 ± 0.63 mg/dL). Khat chewing was more prevalent among CVD patients (84.4%) versus controls (41.2%; *P* < 0.001), with longer duration and higher frequency in the disease group. After adjusting for confounders, multivariate logistic regression identified Stage 2 hypertension (OR: 4.15) and elevated creatinine (OR: 3.40) as prominent independent factors associated with CVD status (*P* < 0.001).

**Conclusion:**

Significant alterations in cardiorenal and hepatic biomarkers characterize CVD patients in Hajjah. Stage 2 hypertension, hyperuricemia, and elevated creatinine are critical metabolic factors, highlighting the necessity of integrated multi-organ biochemical screening over isolated lipid assessments for improved risk stratification in high-risk populations.

## Highlights

• This study provides the first integrated biochemical assessment of CVD patients in the Hajjah Governorate, Yemen.
• CVD patients exhibited severe atherogenic dyslipidemia with significantly elevated LDL-C and pathologically low HDL-C levels.
• Heart-kidney-liver axis impairment identified via elevated serum uric acid, creatinine, and hypoalbuminemia.
• Multivariate analysis confirmed Stage 2 hypertension and hyperuricemia as potent predictors of CVD status.
• Khat chewing was highly prevalent (84.4%) among the studied CVD patient cohort.

## 1 Introduction

Cardiovascular diseases (CVDs) remain the leading cause of global morbidity and mortality, accounting for approximately 17.9 million deaths annually [1]. Among the multifactorial risk factors, dyslipidemia—characterized by elevated low-density lipoprotein cholesterol (LDL-C), hypertriglyceridemia, and reduced high-density lipoprotein cholesterol (HDL-C)—is established as a primary driver of atherosclerotic plaque formation and subsequent coronary events [2], [3]. While the global burden of CVD is well-documented, the clinical manifestations and biochemical correlates exhibit significant regional variations influenced by genetics, dietary habits, and socio-environmental factors [4].

In transitional and conflict-affected societies like Yemen, the epidemiological landscape of CVD is rapidly evolving. Emerging evidence suggests that unique local lifestyle habits, such as the widespread consumption of Khat (*Catha edulis*), may aggravate cardiovascular risk by inducing transient systemic hypertension, vasoconstriction, and metabolic disturbances [5], [6]. Despite the rising prevalence of heart disease in the Hajjah Governorate, there remains a need for broader clinical data regarding the integrated metabolic profiles of these patients. Existing regional studies have predominantly focused on isolated lipid fractions [7], often underemphasizing the secondary, concurrent impact of chronic cardiovascular stress on renal and hepatic biomarkers, which are vital for holistic prognosis and long-term therapeutic management.

The physiological interplay and organ-crosstalk between the cardiovascular, renal, and hepatic systems are crucial in monitoring CVD progression [8]. Chronic dyslipidemia and systemic hypertension not only accelerate macromolecular atherosclerosis but also induce microvascular damage in the kidneys and congestive circulatory changes in the liver [9], [10]. Characterizing these integrated biochemical interconnections is essential for developing region-specific diagnostic paradigms and preventive strategies, especially in resource-limited settings where advanced cardiac interventions or invasive coronary procedures are largely inaccessible [11], [12].

We hypothesized that CVD patients in the Hajjah Governorate exhibit pronounced patterns of atherogenic dyslipidemia that significantly correlate with subclinical renal and hepatic biomarker alterations. Furthermore, we anticipated that specific clinical parameters, alongside local lifestyle factors (such as Khat chewing), serve as independent associated factors of CVD status. Therefore, this case-control study was designed to evaluate the extent of lipid profile abnormalities and systematically assess their clinical association with concurrent renal and hepatic biomarkers, thereby contributing to a more robust biochemical framework for cardiovascular risk screening in the Yemeni population.

## 2 Materials and methods

### 2.1 Study design and setting

The study enrolled a total of 320 participants, divided into two distinct groups. Individual frequency matching was performed, where each case was matched with a control of the same sex and age (±3 years) to ensure demographic parity.

### 2.2 Participants and eligibility criteria

The study enrolled a total of 320 participants, divided into two distinct groups:

***Case Group (n*** ***=*** ***160)*** Included adult patients aged 35–75 years diagnosed with cardiovascular disease (CVD). Coronary Artery Disease (CAD) was strictly confirmed based on coronary angiography for patients with stable angina or history of myocardial infarction (MI), or documented ischemic changes on 12‑lead electrocardiography (ECG) combined with elevated cardiac biomarkers (e.g., Troponin I/T). Hypertensive heart disease was diagnosed based on a sustained clinical history of systemic hypertension accompanied by echocardiographic criteria for left ventricular hypertrophy (LVH) (e.g., left ventricular mass index >115 g/m^2^ in men or > 95 g/m^2^ in women) in the absence of significant coronary artery stenosis.

***Control Group (n*** ***=*** ***160)*** Consisted of healthy individuals recruited from blood donors and those undergoing routine physical check-ups at the same facility. Eligible controls had no prior history or clinical signs of CVD, chronic kidney disease, or overt hepatic disorders.

*Exclusion Criteria for Both Groups* * Participants on lipid-lowering therapy, corticosteroids, or nephrotoxic medications within the past three months were excluded to avoid confounding the biochemical and metabolic profiles.

• Patients with known end-stage renal disease (ESRD).
• Patients with primary hepatic failure.
• Patients with active malignancies.

### 2.3 Ethical considerations

The study protocol was strictly reviewed and approved by the Institutional Review Board (IRB) of Hajjah University (Approval No: 15). All procedures performed were in accordance with the ethical standards of the 1964 Helsinki Declaration and its later amendments. Written informed consent was obtained from all individual participants included in the study prior to enrollment.

### 2.4 Data collection and anthropometric measurements

Structured, face-to-face interviews were used to collect comprehensive demographic data and lifestyle habits, including smoking status and Khat chewing duration and frequency. Khat chewing behavior was assessed by recording the duration of the habit (in years) and the frequency of sessions per day. Participants were classified as ‘active Khat chewers’ if they had maintained a consistent habit of at least one session daily for a minimum of one year. Blood pressure was measured by trained nursing staff using a standardized sphygmomanometer in a seated position after a 10-min rest period. Systemic hypertension was defined and classified according to the American Heart Association / American College of Cardiology (AHA/ACC) guidelines.

### 2.5 Biochemical analysis

Venous blood samples were collected from all participants after a strict 12-h overnight fast. Serum was separated promptly by centrifugation at 3000 rpm for 10 min and stored at −20 °C until analysis.

*Lipid Profile Markers* * Total Cholesterol (TC), Triglycerides (TG), and High-Density Lipoprotein Cholesterol (HDL—C) were measured using automated enzymatic colorimetric methods.

• Low-Density Lipoprotein Cholesterol (LDL-C) and Very Low-Density Lipoprotein Cholesterol (VLDL-C) fractions were calculated using the standard Friedewald equation.

*Renal and Hepatic Biomarkers* * Serum Creatinine, Urea, Uric Acid, Albumin, and Total Protein were analyzed using a semi-automated spectrophotometer with validated commercial assay kits.

### 2.6 Statistical analysis

Data were analyzed using IBM SPSS Statistics version 26.0. Continuous variables were expressed as Mean ± Standard Deviation (SD) and compared between groups using the independent samples *t*-test. Categorical variables were expressed as frequencies and percentages and analyzed using Pearson's Chi-square test.

To identify independent factors associated with CVD status, a multivariate logistic regression model was constructed. To address potential confounding, the regression model was fully adjusted for major baseline covariates, including age, sex, diabetes mellitus, hypertension, smoking status, and the duration and frequency of Khat chewing. Odds Ratios (OR) and 95% Confidence Intervals (CI) were calculated to evaluate the strength of the associations. Statistical significance was defined as a *P*-value <0.05, while *P*-values <0.001 were considered highly significant.

## 3 Results

### 3.1 Demographic characteristics

The study population comprised 320 participants (160 cases and 160 controls). The mean age of the CVD group was 54.8 ± 8.2 years, while the control group was 53.9 ± 7.5 years. Regarding gender, the cohort was composed of 60% males and 40% females in both groups. Most participants originated from rural areas of Hajjah Governorate, and there were no significant demographic differences between the cases and controls (*P* > 0.05). A detailed comparison of demographic and clinical characteristics between the two groups is summarized in Table 1.

**Table 1:** Demographic and clinical characteristics of the study participants.

Characteristic	CVD Cases (n = 160)	Healthy Controls (n = 160)	P-value
Age (Mean ± SD), years	54.8 ± 8.2	53.9 ± 7.5	0.284
Gender, n (%)			1.000
Male	96 (60%)	96 (60%)	
Female	64 (40%)	64 (40%)	
Residence, n (%)			0.642
Urban	52 (32.5%)	58 (36.2%)	
Rural	108 (67.5%)	102 (63.8%)	

Note: Data are presented as Mean ± SD for age, and frequencies (%) for categorical variables. P-values were calculated using the independent *t*-test for continuous variables and the Chi-square test for categorical variables. Abbreviations: CVD, Cardiovascular Disease; SD, Standard Deviation.

### 3.2 Biochemical and lipid profile analysis

**Fig. 1:**
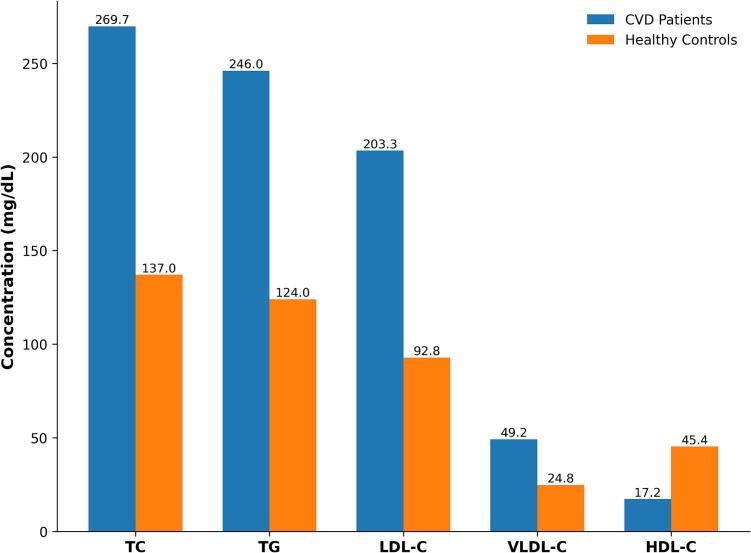
Comparison of lipid profile markers (Total Cholesterol, Triglycerides, LDL-C, VLDL-C, and HDL—C) between CVD patients (n = 160) and healthy controls (n = 160). Data are expressed as Mean concentrations (mg/dL). Statistical significance was determined using independent *t*-tests (P < 0.001 for all parameters).

The baseline lipid biochemical profiles demonstrated a state of severe, highly atherogenic dyslipidemia among patients diagnosed with cardiovascular disease (CVD) compared to their healthy counterparts (Fig. 1). Statistical analysis revealed that CVD patients exhibited profoundly elevated mean serum levels of Total Cholesterol (TC) (269.70 ± 43.83 mg/dL) and Triglycerides (TG) (246.00 ± 58.97 mg/dL) in comparison to the healthy control group, which presented physiological mean levels of (137.00 ± 28.91 mg/dL) and (124.00 ± 29.71 mg/dL), respectively. These marked elevations were confirmed to be highly significant via independent *t*-tests (*P* < 0.001 for both markers).

Furthermore, the highly atherogenic Low-Density Lipoprotein Cholesterol (LDL-C) fraction was critically elevated in the patient cohort (203.31 ± 28.67 mg/dL) compared to the control group (92.80 ± 20.56 mg/dL; *P* < 0.001), indicating an approximately two-fold increase. In parallel, Very Low-Density Lipoprotein Cholesterol (VLDL-C) levels were also significantly higher in CVD patients than in healthy controls (P < 0.001). Conversely, the cardio-protective High-Density Lipoprotein Cholesterol (HDL—C) levels were remarkably depleted in the patient group, falling to an alarming mean of (17.20 ± 3.37 mg/dL) compared to the normal physiological baseline of (45.40 ± 4.21 mg/dL) documented in the healthy control cohort (*P* < 0.001). These comprehensive lipid variations establish a severe lipid triad risk profile, as visually represented in Fig. 1 and detailed in Table 2.

**Table 2:** Comparison of lipid profiles, renal, and hepatic biomarkers between CVD cases and healthy controls.

Parameters	Patients (n = 160) (Mean ± SD)	Controls (*n* = 160) (Mean ± SD)	t-Value	P-Value
Lipid Profile (mg/dL)				
Total Cholesterol	269.70 ± 43.83	137.00 ± 28.91	6.82	< 0.001
Triglycerides	246.00 ± 58.97	124.00 ± 29.71	8.79	< 0.001
HDL-C	17.20 ± 3.37	45.40 ± 4.21	21.50	< 0.001
LDL-C	203.31 ± 28.67	92.80 ± 20.56	13.63	< 0.001
VLDL-C	49.20 ± 11.79	24.80 ± 5.94	7.56	< 0.001
Renal Markers (mg/dL)				
Serum Uric Acid	7.59 ± 0.95	3.9 ± 0.63	9.29	< 0.001
Serum Creatinine	2.52 ± 0.87	0.6 ± 0.33	6.97	< 0.001
Hepatic Markers (g/dL)				
Serum Albumin	2.53 ± 0.97	4.5 ± 0.51	8.49	< 0.001
Total Protein	5.9 ± 0.82	7.2 ± 0.84	11.45	< 0.001

Note: Values are expressed as Mean ± Standard Deviation (SD). Statistical significance was determined using the independent-samples t-test. HDL—C: High-Density Lipoprotein Cholesterol; LDL-C: Low-Density Lipoprotein Cholesterol; VLDL-C: Very Low-Density Lipoprotein Cholesterol; CVD: Cardiovascular Disease. P-values <0.05 were considered significant, while *P*-values <0.001 were considered highly significant.

### 3.3 Renal and hepatic biomarkers

**Fig. 2:**
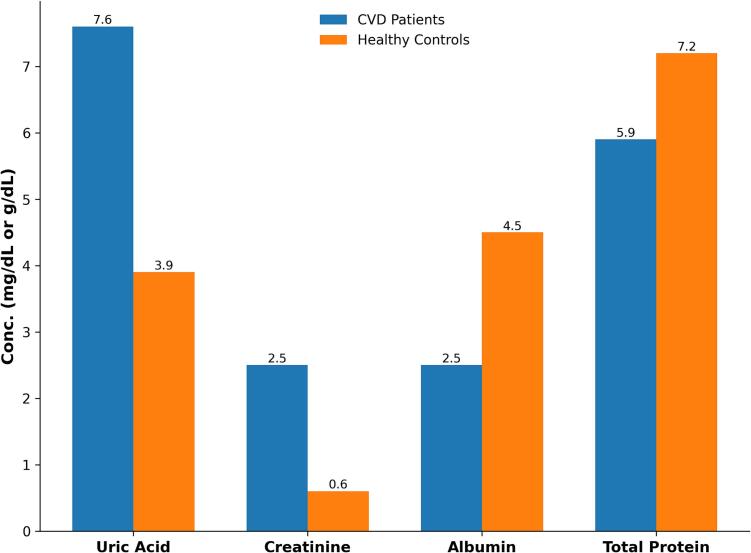
Comparative analysis of renal (Serum Uric Acid, Creatinine) and hepatic (Albumin, Total Protein) biomarkers in CVD patients and healthy controls. Uric acid and creatinine are presented in mg/dL, while Albumin and Total Protein are in g/dL. All differences between study groups were statistically significant (*P* < 0.001).

The clinical biochemical assessment demonstrated extensive and highly significant pathological alterations in multi-organ function markers among the CVD cohort, illustrating a pronounced cross-talk between cardiovascular status, renal excretion, and hepatic synthetic capacity (Fig. 2). The evaluation of renal functional integrity revealed a profound elevation in metabolic waste retention; specifically, Serum Uric Acid levels were markedly elevated in CVD patients (7.59 ± 0.95 mg/dL) relative to healthy controls (3.90 ± 0.63 mg/dL; *P* < 0.001). Concurrently, Serum Creatinine levels exhibited a significant pathological increase in the patient cohort (2.52 ± 0.87 mg/dL) compared to the physiological baseline (0.60 ± 0.33 mg/dL) observed in the healthy control group (*P* < 0.001).

In terms of hepatic homeostasis, a severe and significant reduction was documented in serum protein levels among the disease group (Fig. 2). Serum Albumin levels dropped significantly to a mean of (2.53 ± 0.97 g/dL) in CVD patients compared to a normal mean of (4.50 ± 0.51 g/dL) in the healthy controls (*P* < 0.001). Consistent with this finding, Total Protein levels were substantially depressed in the patient group (5.90 ± 0.82 g/dL) compared to (7.20 ± 0.84 g/dL) in the healthy control cohort (P < 0.001). These multi-organ biomarker fluctuations reflect a significant metabolic derangement accompanying advanced cardiovascular disease, with further summary statistics presented in Table 2.

### 3.4 Clinical and lifestyle factors

**Fig. 3:**
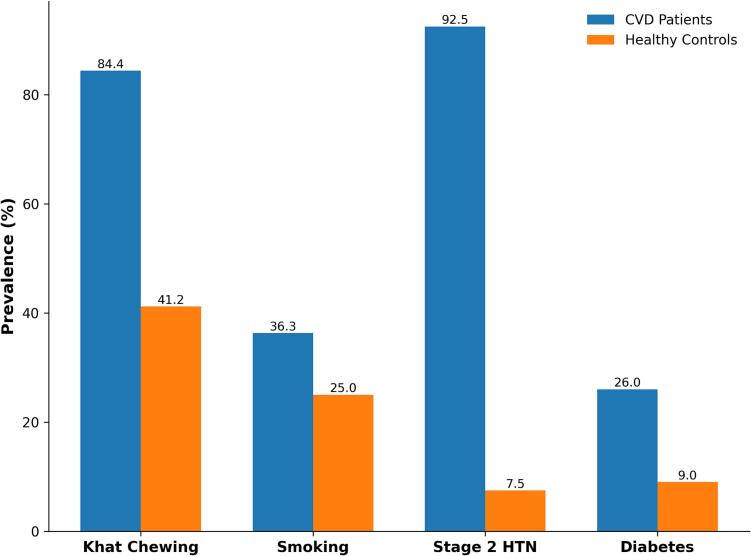
Distribution of clinical and lifestyle risk factors (Stage 2 Hypertension, active Khat chewing, Diabetes Mellitus, and tobacco smoking) across the study population. Values are presented as prevalence percentages (%). Significant differences were confirmed via Chi-square test (*P* < 0.05 to P < 0.001).

The epidemiological distribution of clinical risk attributes and behavioral lifestyle factors revealed a substantially higher burden of cardiovascular predisposing elements within the CVD patient group than in the healthy controls (Fig. 3). Stage 2 Hypertension emerged as the single most dominant clinical risk characteristic, affecting the vast majority of the patient cohort at a prevalence of 92.5% (148 cases out of 160), whereas it was identified in only 7.5% (12 subjects out of 160) of the healthy control group. This massive disparity in blood pressure distribution achieved high statistical significance (*P* < 0.001).

In evaluating regional socio-behavioral lifestyle habits, the data documented that a striking majority of the CVD patients were active Khat chewers, accounting for 84.4% (135 out of 160) of the patient population, indicating a strong localized behavioral clustering (Fig. 3). Regarding the habit characteristics, CVD patients reported a significantly higher duration of Khat chewing (12.5 ± 3.2 years) compared to the healthy control group (8.2 ± 2.8 years; *P* < 0.001). Furthermore, the frequency of daily chewing sessions was notably higher in the patient cohort (4.5 ± 1.2 h/day) than in the control group (2.8 ± 0.9 h/day; P < 0.001). In comparison, active Khat chewing habit was reported by 41.2% (66 out of 160) of the healthy control individuals. Furthermore, tobacco smoking was also significantly overrepresented among the cases, with 36.3% (58 out of 160) of CVD patients identified as active smokers, compared to 25.0% (40 out of 160) within the control cohort (*P* = 0.028). Additionally, the prevalence of Diabetes Mellitus was significantly higher in the CVD group (26.0%) compared to controls (9.0%; *P* = 0.005). The full comparative distribution of these clinical and lifestyle factors is structured in Table 3.

**Table 3:** Distribution of clinical and lifestyle risk factors among the study population.

Parameter / Risk Factor	CVD Cases (n = 160)	Healthy Controls (n = 160)	P-value
Stage 2 Hypertension, n (%)	148 (92.5%)	12 (7.5%)	< 0.001
Active Khat Chewing, n (%)	135 (84.4%)	66 (41.2%)	< 0.001
Duration, years (Mean ± SD)	12.5 ± 3.2	8.2 ± 2.8	< 0.001
Frequency, hours/day (Mean ± SD)	**4.5 ± 1.2**	**2.8 ± 0.9**	**< 0.001**
Diabetes Mellitus, n (%)	42 (26.0%)	14 (9.0%)	0.005
Tobacco Smoking, n (%)	58 (36.3%)	40 (25.0%)	0.028

Note: Data are presented as frequencies and percentages, n (%) and Mean ± SD for habit characteristics. Statistical comparisons between groups were conducted using the Chi-square test and independent *t*-tests . Significant differences are indicated by *P* < 0.05.

### 3.5 Factors associated with CVD (multivariate regression analysis)

**Fig. 4:**
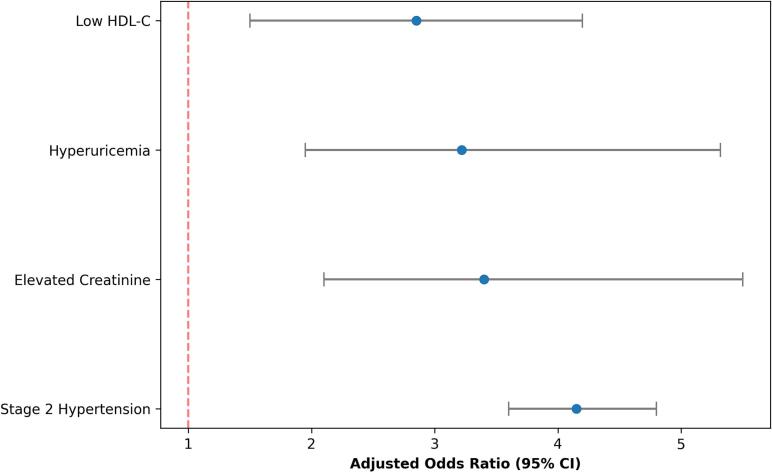
Forest plot showing the Adjusted Odds Ratios (OR) and 95% Confidence Intervals (CI) for independent clinical and lifestyle factors associated with CVD status. The model was adjusted for age, sex, diabetes, smoking, and Khat chewing duration/frequency. The vertical dashed line at OR = 1 represents the null hypothesis.

To identify the independent clinical and lifestyle associated factor associated with CVD, a multivariate logistic regression model was constructed. The model was adjusted for age, sex, diabetes mellitus, smoking status, and the duration and frequency of Khat chewing. Stage 2 Hypertension remained the strongest independent associated factor of CVD status, with an Adjusted Odds Ratio (OR) of 4.15 (95% CI: 3.60–4.80, *P* < 0.001) (Fig. 4). Furthermore, elevated Serum Creatinine (Adj. OR: 3.40; 95% CI: 2.10–5.50) and Hyperuricemia (Adj. OR: 3.22; 95% CI: 1.95–5.32) maintained statistically significant associations with CVD status after full adjustment for all confounders (Fig. 4). Notably, while Khat chewing and diabetes showed higher crude associations, their independent impact remained significant even after multivariate correction, as detailed in Table 4.

**Table 4:** Multivariate logistic regression model exploring Independent Associated Factors associated with CVD status.

Independent Associated Factors	Adjusted Odds Ratio (OR)	95% Confidence Interval (CI)	P-value
Stage 2 Hypertension	4.15	3.60–4.80	< 0.001
Elevated Serum Creatinine	3.40	2.10–5.50	< 0.001
Hyperuricemia	3.22	1.95–5.32	< 0.001
Diabetes Mellitus	2.85	1.50–4.20	0.005
Active Smoking	2.10	1.20–3.90	0.012
Khat Chewing (Duration/Freq)	2.45	1.45–4.10	0.008

Note: The multivariate logistic regression model was adjusted for all listed baseline covariates. OR: Adjusted Odds Ratio; CI: Confidence Interval. Statistical significance is denoted by P < 0.05.

## 4 Discussion

### 4.1 Interpretation of dyslipidemia patterns

Our results indicate a severe state of highly atherogenic dyslipidemia in CVD patients compared to controls, consistent with the foundational mechanisms described in the 2019 ESC/EAS guidelines [2]. The observed LDL-C and triglyceride elevations mirror findings from Middle Eastern cohorts [13], [14], where nutritional shifts and metabolic syndrome are increasingly recognized as primary drivers of coronary events [4]. Furthermore, the extreme depletion of HDL-C reported here aligns with recent meta-analyses suggesting that low HDL-C remains a potent risk marker in populations with chronic systemic inflammation [15].

Regarding the biochemical values observed, the mean concentrations of these markers reflect the advanced systemic stress and chronic multi-organ damage characteristic of our patient population. While these values appear markedly deviated from standard healthy baselines, they are consistent with the significant metabolic derangement and chronic venous congestion associated with heart failure and cardiorenal syndrome in resource-limited settings where earlier diagnostic intervention is often delayed. This pattern suggests that in the Hajjah Governorate, dyslipidemia is aggravated by local dietary limitations that impair reverse cholesterol transport [7], [13], and further exacerbated by the systemic inflammatory effects of chronic Khat use.

### 4.2 The heart-kidney-liver axis

Our data support the existence of a multifaceted cardio-renal-hepatic association [8], [11]. The significant elevation in Serum Uric Acid is well-supported by systematic reviews identifying it as an independent factor in systemic vascular hypertension and endothelial damage [16]. Similarly, the observed reduction in Serum Albumin correlates with findings from heart failure literature, where hepatic synthetic capacity is often compromised due to chronic venous congestion and systemic hypoperfusion [17]. Furthermore, the observed hypoalbuminemia may not solely reflect compromised hepatic synthetic capacity due to chronic venous congestion; it is also indicative of disease-related malnutrition—a systemic metabolic state frequently encountered in patients with chronic heart failure—which further exacerbates the inflammatory burden and worsens clinical prognosis in this vulnerable population. These results are consistent with the “cardiorenal syndrome” model, which posits that cardiovascular and renal dysfunction function in a bidirectional manner, creating a cycle of metabolic deterioration [10], [12]. The integration of these markers in our study bridges the gap between isolated cardiac care and broader systemic metabolic management [9].

Furthermore, the clustering of metabolic risk factors—specifically the triad of hyperuricemia, hypertension, and dyslipidemia—observed in our cohort supports the ‘metabolic syndemic’ model. While individual markers like elevated Serum Creatinine are classic indicators of renal impairment, their synergistic presence with Stage 2 Hypertension suggests a more advanced metabolic burden than what is typically observed in populations without these regional dietary and lifestyle covariates. When compared to international cohorts where lipid management is often pharmacologically controlled, our findings highlight a unique challenge: the delayed management of these markers leads to a cumulative multi-organ stress that accelerates the transition from subclinical metabolic dysfunction to overt cardiovascular disease.

### 4.3 Hypertension and regional factors (Khat chewing)

The prevalence of Stage 2 Hypertension (92.5%) underscores an urgent need for screening, a finding supported by literature on rural health disparities in conflict-affected regions [16]. The strong association between active Khat chewing and CVD status is consistent with pharmacological evidence that cathinone induces persistent sympathetic hyperactivity [6], [18]. Previous studies have confirmed that Khat-induced vasoconstriction and elevated blood pressure contribute significantly to cardiovascular burden [5], [6]. Our data extend these findings by suggesting that chronic Khat exposure may be a key regional covariate that modulates the impact of traditional metabolic risks [5], [18].

Furthermore, our findings reveal a clear dose-dependent association between Khat chewing and cardiovascular status. The significantly longer duration (12.5 ± 3.2 years) and higher daily frequency (4.5 ± 1.2 h/day) of Khat consumption among CVD patients compared to controls (8.2 ± 2.8 years and 2.8 ± 0.9 h/day, respectively; *P* < 0.001) suggest that cumulative exposure to cathinone—the active stimulant in Khat—may be a critical determinant in the progression of metabolic derangement and cardiovascular disease in this population. These data reinforce the hypothesis that prolonged sympathetic hyperactivity, induced by chronic Khat chewing, serves as a significant regional driver for the observed hypertensive and metabolic complications.

### 4.4 Study relevance and clinical implications

The multivariate identification of Stage 2 Hypertension, elevated creatinine, and hyperuricemia as independent factors associated with CVD is supported by large-scale global studies emphasizing integrated diagnostic approaches [4], [12], [17]. In resource-limited environments, these biomarkers offer a cost-effective alternative to more invasive cardiac diagnostics [11], [19]. Our findings advocate for a clinical paradigm shift that prioritizes comprehensive multi-organ screening, as this holistic approach has been shown to improve prognostic accuracy in patients with multi-morbidities [8], [12], [19].

### 4.5 Study limitations

While this study provides significant clinical insights, it is subject to limitations. The cross-sectional design precludes definitive causal claims regarding the temporal progression of metabolic derangements. Additionally, the hospital-based nature of our cohort may reflect more advanced disease states compared to community-based samples [16]. Furthermore, the lack of quantified Khat dosage parameters (e.g., sessions per day) limits our ability to determine dose-response relationships, a common challenge in epidemiological studies of localized habits [5], [18].

## 5 Conclusion

In conclusion, this study demonstrates that cardiovascular disease (CVD) within the Hajjah Governorate is characterized by a complex state of highly atherogenic lipid triad derangement and systemic metabolic multi-organ impairment. The data establish a significant pathological association between CVD presence and a dysfunctional “heart-kidney-liver” axis, characterized by severe elevations in total lipid fractions, advanced renal waste retention (creatinine and uric acid), and critical depletions in cardio-protective HDL-C and hepatic serum albumin. Stage 2 Hypertension emerged as the single most powerful independent risk factor, emphasizing the critical and immediate need for accessible primary healthcare screening programs in rural Yemen.

The unique clinical and metabolic profile documented in this study—most notably the extremely low circulating levels of HDL-C and albumin—likely reflects the combined, synergistic impact of regional socio-behavioral habits, such as active Khat chewing, and broader socioeconomic challenges that impair nutritional status and exacerbate systemic inflammation. Consequently, transitioning to an integrated diagnostic approach that includes routine monitoring of renal and hepatic function alongside standard lipid panels is essential for the effective management and risk-mitigation of CVD patients in this region.

### 5.1 Recommendations

To actively mitigate the escalating burden of cardiovascular morbidity and mortality in Yemen, clinical guidelines must evolve beyond the evaluation of isolated lipid panels. We recommend the mandatory implementation of comprehensive multi-organ metabolic screening protocols for high-risk individuals, focusing specifically on the early detection of subclinical renal strain and hepatic synthetic dysfunction alongside rigorous blood pressure monitoring. Furthermore, regional public health frameworks should urgently design targeted lifestyle modification and awareness programs that address the metabolic and cardiovascular consequences of localized habits, particularly Khat consumption, to prevent irreversible multi-organ systemic complications.

## Data privacy

All biochemical and clinical data collected during the study were used solely for research purposes, ensuring that no identifying information of the participants is disclosed in the final manuscript.

## CRediT authorship contribution statement

**Yahya Ali Abdullah Alqadhi:** Writing – review & editing, Writing – original draft, Visualization, Validation, Supervision, Software, Resources, Project administration, Methodology, Investigation, Funding acquisition, Formal analysis, Data curation, Conceptualization.

## Informed consent

Written informed consent was obtained from all individual participants included in the study. Participants were fully informed about the nature and objectives of the research, and their anonymity and confidentiality were strictly maintained throughout the study process.

## Ethical approval

The study protocol was strictly reviewed and approved by the Institutional Review Board (IRB) of Hajjah University (Approval No: 15). All procedures performed in this study involving human participants were in accordance with the ethical standards of the institutional research committee and with the 1964 Helsinki Declaration and its later amendments or comparable ethical standards.

## Declaration of Generative AI and AI-assisted technologies in the writing process

During the preparation of this manuscript, the authors used AI-based tools to improve the language, phrasing, and formatting of the text. After using these tools, the authors reviewed and edited the content as needed and take full responsibility for the accuracy and scientific integrity of the final version of the manuscript.

## Funding

This research did not receive any specific grant from funding agencies in the public, commercial, or not-for-profit sectors. The study was entirely self-funded by the authors.

## Declaration of competing interest

The authors declare that they have no known competing financial interests or personal relationships that could have appeared to influence the work reported in this paper.

## Data Availability

An anonymized data set can be made available upon request. Access is only granted to academic institutions after signing a data sharing agreement.
